# Stimulation of neoplastic mouse lung cell proliferation by alveolar macrophage-derived, insulin-like growth factor-1 can be blocked by inhibiting MEK and PI3K activation

**DOI:** 10.1186/1476-4598-10-76

**Published:** 2011-06-24

**Authors:** Jason M Fritz, Lori D Dwyer-Nield, Alvin M Malkinson

**Affiliations:** 1 Department of Pharmaceutical Sciences, Skagg School of Pharmacy and Pharmaceutical Sciences, University of Colorado Anschutz Medical Campus, 12850 E. Montview Blvd, C-238 V20-4460, Aurora, CO. 80045, USA

**Keywords:** Lung cancer, macrophages, proliferation, IGF-1, cytokines

## Abstract

**Background:**

Worldwide, lung cancer kills more people than breast, colon and prostate cancer combined. Alterations in macrophage number and function during lung tumorigenesis suggest that these immune effector cells stimulate lung cancer growth. Evidence from cancer models in other tissues suggests that cancer cells actively recruit growth factor-producing macrophages through a reciprocal signaling pathway. While the levels of lung macrophages increase during tumor progression in mouse models of lung cancer, and high pulmonary macrophage content correlates with a poor prognosis in human non-small cell lung cancer, the specific role of alveolar macrophages in lung tumorigenesis is not clear.

**Methods:**

After culturing either an immortalized lung macrophage cell line or primary murine alveolar macrophages from naïve and lung-tumor bearing mice with primary tumor isolates and immortalized cell lines, the effects on epithelial proliferation and cellular kinase activation were determined. Insulin-like growth factor-1 (IGF-1) was quantified by ELISA, and macrophage conditioned media IGF-1 levels manipulated by IL-4 treatment, immuno-depletion and siRNA transfection.

**Results:**

Primary macrophages from both naïve and lung-tumor bearing mice stimulated epithelial cell proliferation. The lungs of tumor-bearing mice contained 3.5-times more IGF-1 than naïve littermates, and media conditioned by freshly isolated tumor-educated macrophages contained more IGF-1 than media conditioned by naïve macrophages; IL-4 stimulated IGF-1 production by both macrophage subsets. The ability of macrophage conditioned media to stimulate neoplastic proliferation correlated with media IGF-1 levels, and recombinant IGF-1 alone was sufficient to induce epithelial proliferation in all cell lines evaluated. Macrophage-conditioned media and IGF-1 stimulated lung tumor cell growth in an additive manner, while EGF had no effect. Macrophage-derived factors increased p-Erk1/2, p-Akt and cyclin D1 levels in neoplastic cells, and the combined inhibition of both MEK and PI3K ablated macrophage-mediated increases in epithelial growth.

**Conclusions:**

Macrophages produce IGF-1 which directly stimulates neoplastic proliferation through Erk and Akt activation. This observation suggests that combining macrophage ablation therapy with IGF-1R, MEK and/or PI3K inhibition could improve therapeutic response in human lung cancer. Exploring macrophage-based intervention could be a fruitful avenue for future research.

## Background

Lung cancer is a worldwide epidemic. In 2009, nearly 160,000 people died from lung cancer in the U.S. alone. The five-year survival rate slightly increased from 13% to 15% over the last 25 years, mainly due to limited early cancer detection and minor improvements in therapy [1]. Non-small cell lung cancer (NSCLC) is the most common form of the disease, and adenocarcinoma (AC) of the distal lung the most frequently diagnosed subtype [2]. Persistent lung inflammation due to cigarette smoke and related pulmonary comorbidities such as chronic obstructive pulmonary disease increases the lifetime risk of developing lung cancer [3], which can be partially alleviated by long-term anti-inflammatory drug therapy [4,5]. Therefore, delineating the causal relationship between inflammation and lung carcinogenesis may lead to earlier diagnosis and more effective treatment.

To understand how chronic lung inflammation promotes the growth of lung cancer, it is important to examine communication between pulmonary epithelial cells and inflammatory effector cells such as alveolar macrophages. Macrophages are the most abundant type of immune cell in a healthy lung [6], and alveolar macrophage numbers increase dramatically as chronic diseases like NSCLC progress [7-9]. Macrophages infiltrate most solid cancers, including NSCLC, and lung cancer patients display an inverse relationship between macrophage infiltration and survival [7,9]. Local environmental stimuli modulate macrophage function, a process referred to as macrophage activation or polarization. Classical macrophage activation arises in response to tissue damage signals, whereas alternative activation is associated with wound healing and cancer progression [10,11]. In experimental mouse models of NSCLC, alveolar macrophages become alternatively activated within weeks of lung tumor initiation [6]. Chemical depletion of macrophages delays lung tumorigenesis, while chemically-induced chronic inflammation greatly increases lung macrophage content and stimulates lung tumor growth [12].

Although the mechanisms by which recruited macrophages contribute to lung AC growth and progression have not been delineated, the reciprocal growth factor interaction between macrophages and breast cancer cells suggests one possibility [13,14]. In mouse models of invasive breast cancer, macrophage-secreted epidermal growth factor (EGF) stimulates growth and migration of mammary tumor cells, which in turn secrete colony stimulating factor-1 (CSF-1) to recruit additional macrophages to the tumor site [13]. This reciprocal growth factor signaling cascade can induce the migration of neoplastic cells from the primary breast tumor site into systemic circulation, dramatically increasing the potential for metastatic colonization [15]. Unlike breast cancer, little is known regarding the contribution of macrophage-derived growth factors to lung cancer growth.

Compared to macrophages in other tissues, the alveolar macrophage is fairly unique due to the monocyte differentiation cytokines present in the lung microenvironment. Specifically, granulocyte-monocyte colony stimulating factor (GM-CSF) is highly expressed while local concentrations of CSF-1 are typically low. High levels of GM-CSF induce the differentiation of blood monocytes into dendritic-like cells, instead of the more traditional macrophage-like fate directed by CSF-1 [9]. Consistent with these observations, alveolar macrophages more closely resemble immature dendritic cells than do macrophages isolated from other tissues [16]. Because of these distinct differences in morphology and function, pulmonary macrophages may stimulate lung cancer proliferation by providing growth factors different than those described in breast and ovarian cancer. While cultured lung AC cells produce several macrophage chemoattractants, including IL-1β and GM-CSF [17,18], there are few reports of any reciprocal growth factor exchange between primary alveolar macrophages and NSCLC [18]. Although the specific factors have not been clearly identified, tumor growth may be stimulated through common downstream signaling mechanisms such as increased Erk1/2 activity, as Erk1/2 is hyper-activated in NSCLC [19,20]. Thus, in addition to identifying lung macrophage-derived tumor growth factors, targeting signaling pathways common to neoplastic growth may also be therapeutically beneficial.

Nearly 25% of NSCLCs contain activating mutations in *KRAS*, resulting in growth stimulation through increased Erk1/2 and Akt activities [21,22]. Kras-mediated activation of extracellular-regulated kinase kinase (MEK) and phosphoinositide-3 kinase (PI3K) directly increases proliferation and cell survival through transcriptional regulation, increased cell cycle progression, and inhibition of pro-apoptotic factors [20,23]. Although Kras signals through multiple downstream effectors, experimental studies have shown that lung tumors containing mutated *Kras *are clearly dependent on cellular kinases such as Erk1/2 and Akt for continued growth and survival [24]. Mutations in *Kras *are sufficient to initiate lung tumorigenesis [25], and chronically high lung macrophage content greatly accelerates the growth and progression of this disease [5,12]. Many growth factors stimulate Erk1/2 and Akt activity in healthy tissues; among these, insulin-like growth factor 1 (IGF-1) is associated with neoplastic growth and expansion [26,27]. In mouse lungs, IGF-1 was originally identified as an alveolar macrophage-derived growth factor [28,29], and increased macrophage IGF-1 production has been observed in models of environmental lung injury [30]. IGF-1 receptor inhibition is currently under intensive clinical investigation, and early reports show therapeutic promise in some NSCLC patients [12,27]. Therefore, IGF-1 could be one candidate by which lung macrophages accelerate the growth of lung tumors.

We sought to determine if chronic inflammation drives lung tumorigenesis, in part, by recruiting and polarizing alveolar macrophages, which in turn produce IGF-1 that directly stimulates neoplastic growth. Since both healthy and tumor-bearing lungs contain dozens of unique resident and infiltrating cell types [31], we co-cultured primary and immortalized mouse lung cells with macrophages, and demonstrated increased epithelial proliferation after exposure to macrophages in a simplified *in vitro *system. Such macrophage co-culture stimulated Erk1/2 and Akt activation, increased cyclin D1 expression, and enhanced the proliferation of neoplastic lung cells; the inhibition of both MEK and PI3K could block this macrophage-augmented tumor cell growth. IGF-1 was detected in lung lavage fluid and macrophage conditioned media, and was significantly elevated in tumor-bearing lungs and tumor-educated macrophage-conditioned media. Our findings demonstrate that macrophages recruited to the chronically inflamed lung have an enhanced ability to directly augment neoplastic growth, suggesting that specifically targeting tumor-associated macrophages, in addition to macrophage-derived growth factors, may be beneficial for future cancer therapy.

## Results

### Macrophage conditioned media profoundly stimulates the anchorage-independent growth of lung tumor cells

**Figure 1:**
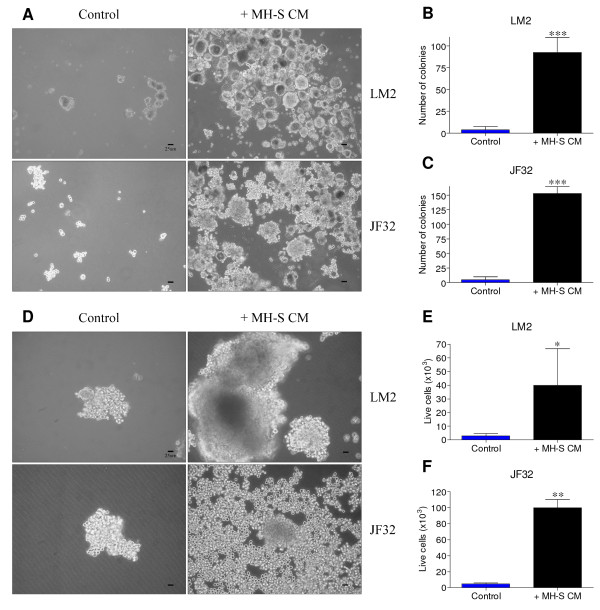
**Anchorage-independent neoplastic lung cell line proliferation is augmented by macrophage conditioned media**. *A: *Neoplastic LM2 or JF32 cells were cultured on 0.75% agarose in serum-free media containing 0.5% BSA (Control), or in similar media conditioned by MH-S macrophages (+ MH-S CM). *B-C: *LM2 or JF32 colonies from (A) were quantified under 20× magnification, and means + SD from duplicate wells plotted from three independent experiments (n = 6). *D: *Neoplastic LM2 or JF32 cells were cultured on ultra-low attachment culture plates, and treated as above. *E-F: *LM2 or JF32 cells from (D) were quantified and mean + SD of live cell numbers plotted from three independent experiments (n = 3). * *P *< 0.05, ** *P *< 0.01 and *** *P *< 0.001 versus control by student's unpaired t-test.

Despite the correlation between lung macrophage content and lung tumor growth, the direct contribution of alveolar macrophages to lung tumor growth is unclear [6,7,13]. Media conditioned by an immortalized lung macrophage cell line, MH-S, has been previously reported to stimulate the migration of lung epithelial cells harboring *Kras *mutations [18]. To determine if MH-S conditioned media directly stimulates neoplastic growth, we first evaluated neoplastic colony formation and cell number after long-term conditioned media exposure. In both the classic model of anchorage-independent neoplastic growth on soft agar (Figure 1A-C), and colonization on new ultra-low adherence, neutrally-charged plastic (Figure 1D-F), macrophage-conditioned media potently stimulated the proliferation of two *Kras *mutant lung tumor-derived cell lines (LM2 and JF32). Thus, macrophages secrete soluble molecules capable of greatly stimulating neoplastic colony formation and proliferation *in vitro*, which may shed light on the role of macrophage recruitment to lung cancer *in vivo*.

### Naïve and tumor-educated primary macrophage co-culture stimulates the proliferation of neoplastic and non-neoplastic pulmonary epithelial cells

**Figure 2:**
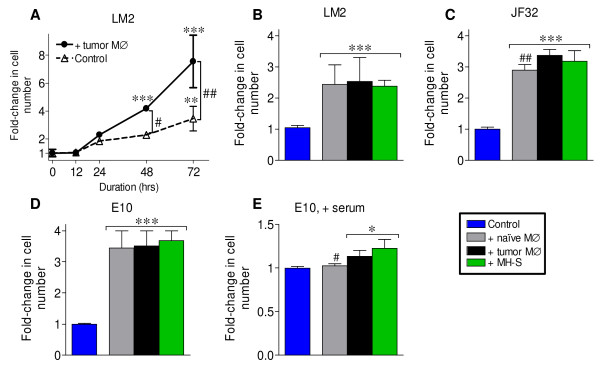
**Lung cell proliferation is accelerated by alveolar macrophage co-culture**. *A: *Neoplastic LM2 cells were cultured alone (Δ) or with primary BAL macrophages from tumor-bearing mice (●) in serum-free media. *B-D: (B) *LM2, *(C) *JF32 and *(D) *E10 cell lines were co-cultured with BAL macrophages (MØ) from naïve (grey bars) or tumor-bearing (black bars) mice, or the MH-S macrophage cell line (green bars) for 72 hrs in serum-free media. *E: *E10 cells were co-cultured as in *(D)*, but in media containing 10% serum. Epithelial cells cultured in the absence of macrophages were controls (blue bars). Proliferation was determined as described, and mean ± SD pooled from at least 3 independent experiments plotted as fold-change from control (normalized to 1). * *P *< 0.05, ** *P *< 0.01 and *** *P *< 0.001 versus control; # *P *< 0.01, ## *P *< 0.001 between *(A) *conditions at the indicated time points, or *(C, E) *between proliferation induced by primary macrophage source (naïve vs. tumor-bearing mice).

The relative ability of naïve vs. tumor-educated alveolar macrophages to directly stimulate lung epithelial cell proliferation not been reported. To determine if macrophages from the lungs of tumor-bearing mice could directly stimulate neoplastic cell growth in a co-culture system, neoplastic LM2 cells were co-cultured with bronchoalveolar lavage (BAL) macrophages (MØ) isolated from tumor-bearing mice, and monolayer growth was assessed (Figure 2A). Growth in standard tissue culture conditions measures proliferation *per se*, and not cell motility or the requirement for solid support, and permits the evaluation of non-neoplastic epithelial cells which do not proliferate in anchorage-independent systems. LM2 cell number significantly increased with BAL macrophage co-culture at 48 (2.3 vs. 4.1-fold) and 72 hrs (3.5 vs. 7.5-fold) (Figure 2A). As 72 hrs of macrophage co-culture resulted in ≥ 2-times more tumor cells, this time point was used in subsequent experiments. To determine if tumor-educated macrophages stimulated neoplastic growth more effectively than naïve, BAL macrophages from either naïve or tumor-bearing mice were co-cultured with neoplastic LM2 (Figure 2B) and JF32 (Figure 2C) cells. LM2 growth was equally stimulated by both naïve and tumor-educated BAL macrophages, while the growth of JF32 cells was enhanced slightly upon co-culture with tumor-educated BAL macrophages (Figure 2C). To determine if primary alveolar macrophages also stimulated the proliferation of non-tumor cells, the non-neoplastic E10 cell line was co-cultured with naïve and tumor-educated BAL macrophages. Both macrophage types increased E10 cell number 3.5-fold (Figure 2D) when maintained in serum-free conditions; only tumor-educated macrophages stimulated E10 proliferation when cultured in the presence of serum (Figure 2E). Both types of primary macrophages equally stimulated LM2 proliferation in the presence of serum, though the magnitude was reduced when compared to serum-free co-culture (data not shown).

To determine if MH-S macrophages could recapitulate the effects of primary alveolar macrophages in this *in vitro *model, we co-cultured MH-S macrophages with both neoplastic and non-neoplastic lung epithelial cells. MH-S co-culture increased the growth rate of all pulmonary epithelial cell lines similar to co-culture with tumor-educated BAL macrophages (Figure 2B-E). These results indicate that primary lung macrophages produce diffusible signals which can augment the proliferation of both non-neoplastic and neoplastic cells *in vitro*. Further, we observed that *in vivo *tumor education of primary lung macrophages slightly enhances this ability to stimulate epithelial proliferation, an effect similar to co-culture with MH-S macrophages.

### Macrophage co-culture stimulates epithelial proliferation through kinase activation

**Figure 3:**
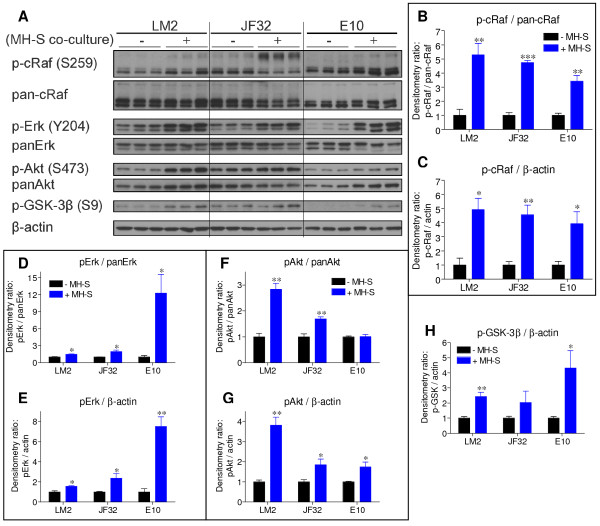
**MH-S co-culture increases activation of growth-associated kinases**. *A: *LM2, JF32 and E10 cells were plated in triplicate, and cultured alone (-) or with MH-S macrophages (+). Protein homogenates from whole cell lysates were probed for expression of phospho-cRaf (p-cRaf), total c-Raf (pan-cRaf), phospho-Erk1/2 (p-Erk), total Erk1/2 (panErk), phospho-Akt (p-Akt), total Akt (panAkt), phospho-GSK-3β and β-actin. *B-H: *Band densitometry was determined as described, and data plotted as percent induction normalized to control intensity. Densitometry presented as mean + SEM from n = 3 replicates combined, and is representative of 3 independent experiments. * *P *< 0.05, ** *P *< 0.01 versus control culture (-MH-S) by unpaired t-test.

Since MH-S macrophages and tumor-educated primary macrophages stimulated epithelial proliferation to a similar degree, MH-S macrophages were used to elucidate the mechanisms of increased epithelial proliferation. Because Kras pathways are commonly hyper-activated in lung tumorigenesis [22,23], and the tumorigenic lines examined herein contain *Kras *mutations, activities of downstream mediators Erk and Akt were examined. Cytosolic Raf functionally links the Erk and Akt pathways; activated Akt can phosphorylate cRaf at S259, placing Erk regulation downstream of Akt activation [32,33]. MH-S co-culture stimulated cRaf phosphorylation at S259 in all three cell lines, resulting in significantly higher levels of p-cRaf (Figure 3A-C). The smaller (~74 kDa) p-cRaf isoform was most highly abundant and its phosphorylation significantly increased with macrophage co-culture in the LM2 and E10 cells, but a larger (~100 kDa) isoform was heavily phosphorylated at the expense of the 74 kDa isoform in neoplastic JF32 cells (Figure 3A). The 74 kDa isoform was the most abundant in total cRaf immunoblots from all three cell lines.

MH-S co-culture significantly increased the levels of active Erk1/2 (p-Erk) in LM2 and JF32 cells, as well as non-neoplastic E10 cells, when normalized either to total Erk (panErk) or β-actin levels (Figure 3A, D and 3E), which correlates with the observed increases in proliferation (Figure 2). E10 cells expressed lower basal p-Erk/panErk vs. the neoplastic cell lines, consistent with previous observations [21]. Total Erk remained unchanged in both neoplastic cell lines, while macrophage co-culture caused Erk2 (42 kDa) to nearly disappear in the E10 cells, with little effect on Erk1 (Figure 3A, D and 3E). Activated Akt (p-Akt) levels rose significantly in both neoplastic cell lines when normalized to either total Akt (panAkt) or β-actin, but macrophage co-culture caused both p-Akt and panAkt levels to rise to similar extents in E10 cells (Figure 3A and 3F). When p-Akt was normalized to panAkt expression, there was no change in E10 cells with MH-S co-culture (Figure 3F). Total Akt expression increased slightly in LM2 cells but decreased in JF32 cells (Figure 3A). When normalized to β-actin, p-Akt levels significantly increased upon MH-S co-culture in all three cell lines (Figure 3A and 3G).

Increased p-S473 Akt content suggests increased enzymatic activity, which can be confirmed by enhanced phosphorylation of downstream substrates. To determine if macrophage co-culture increases Akt activity, we measured levels of p-GSK-3β, a known target of Akt [32]. Consistent with the elevation in p-Akt, MH-S co-culture significantly increased p-GSK-3β in both LM2 and E10 cells and trended towards an increase in JF32 cells (Figure 3A and 3H); panGSK-3β levels were unchanged (data not shown). Phospho-S259 cRaf is another measure of Akt activity, and p-cRaf levels increased in all three cell lines with macrophage co-culture (Figure 3A-C). Together, the observed increases in epithelial proliferation and the known roles for Erk and Akt in neoplastic lung cell division suggest that macrophage co-culture stimulates lung cell proliferation through increased Erk and Akt activity [34].

### Combined inhibition of MEK and PI3K abrogates macrophage stimulation of neoplastic growth

**Figure 4:**
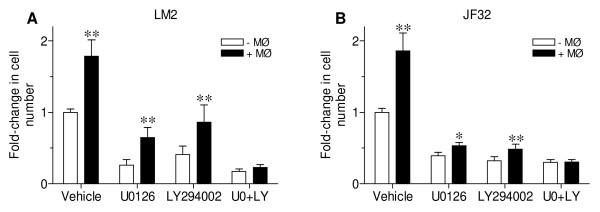
**Macrophage co-culture stimulated neoplastic proliferation can be blocked by combined kinase inhibition**. *A-B: *Neoplastic *(A) *LM2 or *(B) *JF32 cells were cultured alone (-MØ, white bars) or with primary BAL macrophages (+MØ, black bars). Co-culture media was supplemented with 0.05% DMSO (vehicle), 5 μM U0126 (U0) and/or 10 μM LY294002 (LY). Proliferation was determined by MTS and mean + SD plotted as fold-change from vehicle control. Data was pooled from 3 independent experiments. * *P *< 0.05 and ** *P *< 0.001 versus-MØ control for each treatment by 2-way ANOVA.

Erk and Akt regulate both proliferation and resistance to apoptotic cell death, are more active in lung tumors than in normal tissue [21,35], and were activated with macrophage co-culture. Since combined MEK and PI3K inhibition slowed mutant *Kras-*driven lung tumor growth *in vivo *[25], we determined whether selective inhibition of MEK and PI3K affected macrophage-stimulated proliferation in these *Kras *mutant lung tumor cell lines. Selective inhibition of either MEK (by U0126) or PI3K (by LY294002) significantly decreased basal proliferation, and blocked growth stimulated by macrophage co-culture to different extents in LM2 and JF32 cells (Figure 4A and 4B, respectively). Only the combined inhibition of both kinases ablated the stimulatory effect of macrophage co-culture on neoplastic proliferation (U0 + LY, Figure 4). Kinase inhibitors were applied at concentrations reported to be cytostatic and not cytotoxic [34,36,37], and none of these treatments significantly increased LM2 or JF32 cell death (data not shown). These results suggest that both the MEK and PI3K pathways must be blocked to effectively inhibit macrophage-stimulated neoplastic growth.

### Macrophage conditioned media contains 3-10 kDa factors which stimulate neoplastic proliferation

**Figure 5:**
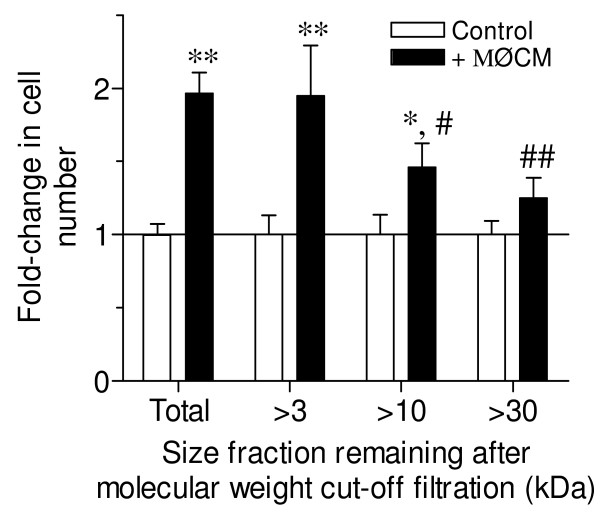
**3-10 kDa molecules in MØCM stimulate epithelial proliferation**. Neoplastic LM2 cells were cultured for 72 hrs after the addition of fresh media (Control, white bars) or MØCM (+MØCM, black bars), separated by size (kDa) into the fractions indicated. Relative cell number was determined by MTS and mean + SD plotted as fold-change from control cultures, normalized for each size fraction. Data was pooled from n = 3 independent experiments. * *P *< 0.01 and ** *P *< 0.001 versus control within each fraction group, and # *P *< 0.01, ## *P *< 0.001 versus total (unfractionated) MØCM by 2-way ANOVA.

Macrophages produce numerous cytokines, eicosanoids and other soluble factors depending upon tissue location and environmental stimuli [4,18], any number of which could be responsible for the observed neoplastic growth stimulation described above. Media conditioned by primary BAL macrophages (MØCM) stimulated the proliferation of LM2 cells, albeit to a lesser extent than primary macrophage co-culture (Figure 5 "Total" vs. Figure 2B). When size-fractionated MØCM was added to LM2 cells, molecules between 3 and 10 kDa stimulated LM2 growth to the greatest extent (Figure 5). Thus, factors of this size mediated the majority of MØCM effects on LM2 growth. Alveolar macrophages produce numerous growth factors in this size range, including IGF-1, GM-CSF and EGF [11,18]. To further narrow down the list of possible candidates, an *in silico *analysis was performed for each fraction size as described in Materials and Methods. The resulting data points were separately fit for each fraction size to the general equation *y *= *y*_0 _+ *a*(1-*e^-bx^*) as described, with regression r^2 ^= 0.997, 0.842 and 0.918 for the > 3, > 10 and > 30 kDa fractions, respectively. From regression analysis, the responsible factor(s) appeared to be 7.23-10.8 kDa in size, suggesting that growth factors such as IGF-1 (7.5 kDa) may be responsible for the MØCM-stimulated neoplastic proliferation.

### Macrophage-conditioned media IGF-1 levels correlate to effects on neoplastic proliferation

**Figure 6:**
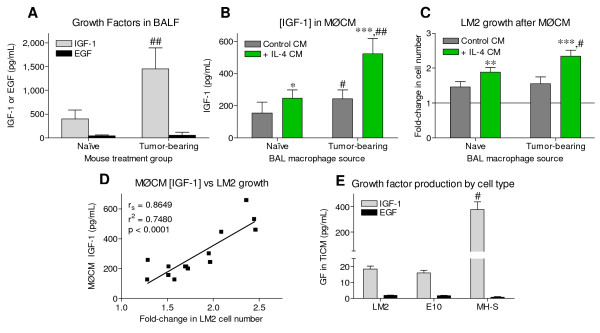
**LM2 proliferation correlates to the IGF-1 levels in macrophage conditioned media**. *A: *IGF-1 and EGF levels in BALF from naïve or tumor-bearing animals, determined by ELISA. *B: *IGF-1 levels in media conditioned by untreated or IL-4 stimulated macrophages from naïve or tumor-bearing animals. *C: *Fold-change in LM2 cell number after addition of MØCM produced in (B), with untreated control LM2 cell numbers normalized to 1 (not shown). *D: *Pearson correlation and linear regression of MØCM IGF-1 levels vs. the fold-change in LM2 cell number after MØCM addition, *P *< 0.001. *E: *IGF-1 and EGF levels in cell line-conditioned media. Cell number was determined as described, and mean + SD plotted as fold-change from control. BALF IGF-1 levels in (A) from n = 12 mice per condition, and *in vitro *data (B-E) was pooled from at least 3 independent experiments. * *P *< 0.05, ** *P *< 0.01, *** *P *< 0.001 versus control, and # *P *< 0.01, ## *P *< 0.001 versus similarly treated naïve groups (A-C) or epithelial cell lines (E) by 2-way ANOVA.

IGF-1 has a well-established role in the metastasis of cancer cells *in vivo*, as well as stimulating growth *in vitro *[27], and alveolar macrophages produce high levels of IGF-1 in response to quartz dust-induced lung injury [30]. While alveolar macrophages are an important component of the chronic inflammatory milieu responsible for promoting lung tumorigenesis, IGF-1 has not been examined as a possible connection between macrophage recruitment and lung cancer progression. BALF from tumor-bearing lungs contained 3.5-times more IGF-1 than BALF from naïve mice, while EGF levels were unchanged (Figure 6A). Even after normalizing to total BALF protein levels, BALF IGF-1 was significantly higher in tumor-bearing animals than naïve controls (1.81 ± 0.33 vs. 0.95 ± 0.36 pg IGF-1/ug BALF protein, respectively, *P *< 0.01, mean ± SD), suggesting that more IGF-1 is produced in the lungs of tumor-bearing mice.

Measurement of IGF-1 levels in MØCM from primary naïve and tumor-educated BAL macrophages showed that tumor-educated macrophages produced significantly more IGF-1 than naïve macrophages (Figure 6B, grey bars). IL-4 potently stimulates alternative macrophage activation, and is more abundant in tumor-bearing lungs than naïve [38]. Alternative macrophage polarization is associated with tumorigenesis [6] and increased macrophage IGF-1 production [39]. Therefore, IL-4 was added to wells containing primary naïve and tumor-educated BAL macrophages to determine if alternative activation could increase IGF-1 production in either macrophage group. Both naïve and tumor-educated macrophages produced significantly more IGF-1 after IL-4 treatment; tumor-educated macrophages more than doubled IGF-1 output compared to naïve samples (Figure 6B, green bars). MH-S macrophages produced 20-times more IGF-1 than either non-neoplastic or neoplastic lung cell lines, and all three cell lines produced only trace amounts (< 2 pg/mL) of EGF (Figure 6E).

In order to determine whether the growth effects of MØCM from samples generated in Figure 6B correlated with their IGF-1 content, MØCM was added to neoplastic LM2 cells. IL-4 stimulated naïve and tumor-educated MØCM significantly augmented LM2 proliferation (Figure 6C, green bars), with IL-4 treated tumor-educated MØCM being the most potent. MØCM from untreated tumor-educated macrophages did not stimulate LM2 growth significantly more than untreated naïve MØCM (Figure 6C, grey bars), corresponding to previous co-culture results (Figure 2B). As the growth-stimulating ability of MØCM appeared to correlate to media IGF-1 levels, the levels of IGF-1 present were plotted against the fold-change in LM2 cell number after MØCM addition (Figure 6D). The correlation between IGF-1 levels and neoplastic growth stimulation was highly significant (p < 0.001), indicating that MØCM IGF-1 levels were directly related to the ability of MØCM to stimulate neoplastic proliferation.

### IGF-1 stimulates lung epithelial cell proliferation and is additive with MØCM

**Figure 7:**
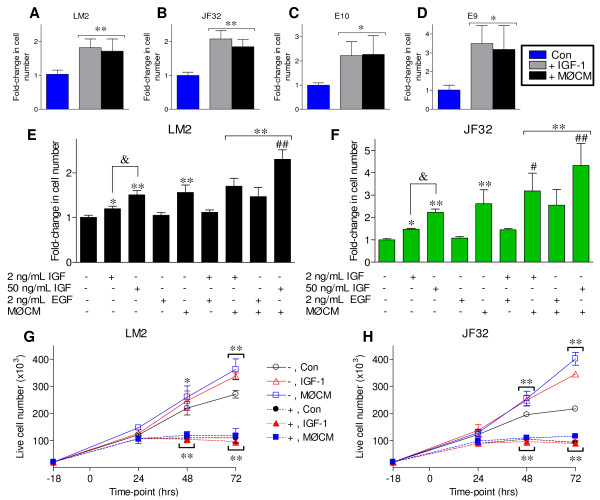
**Neoplastic proliferation stimulated by both IGF-1 and MØCM is additive, but unaffected by EGF**. *A-D: (A) *LM2, *(B) *JF32, *(C) *E10 cells and *(D) *E9 cells were cultured with fresh serum-free media (Con), 50 ng/mL mIGF-1 (+ IGF-1) or media conditioned by MH-S macrophages (+ MØCM). Cell number was determined by MTS and mean + SD plotted as fold-change from control. *E-F: (E) *LM2 or *(F) *JF32 cells were cultured in serum-free media containing 0.5% BSA (control), with growth factors or MØCM. *G-H: (G) *LM2 or *(H) *JF32 live cell number was determined by hemocytometer after culture with or without NVP-AEW541 (- or +, respectively), and 50 ng/mL IGF-1 or MØCM as described above. Growth stimuli were added at 0 hrs, which was 18 hrs after cell plating. Data was pooled from at least 3 independent experiments with all conditions assayed in triplicate, and mean + SD plotted as fold-change from untreated controls (normalized to 1; A-F). * *P *< 0.05 and ** *P *< 0.001 versus control (or "-, Con"), &*P *< 0.001 vs. the 2 ng/mL IGF-1 group and # *P *< 0.05, ## *P *< 0.001 versus MØCM single treatment by 1-way ANOVA.

While IGF-1 levels correlated strongly with the ability of MØCM to stimulate neoplastic growth, IGF-1 induced proliferation of these non-neoplastic and neoplastic mouse lung cell lines has not been demonstrated. Recombinant mouse IGF-1 or MH-S macrophage-conditioned media was sufficient to stimulate the proliferation of neoplastic LM2, JF32 and E9 cells and non-neoplastic E10 cells (Figure 7A-D). The degree of growth stimulated by 50 ng/mL IGF-1 was similar to that of MØCM in each line (Figure 7A-D). These results confirm that IGF-1 alone can stimulate the growth of long-established neoplastic and non-neoplastic cell lines, as well as cells isolated more recently from primary mouse lung tumors (JF32), consistent with previous reports on human cancer cell lines [27]. In order to determine any relevant role of EGFR in mediating macrophage-stimulated tumor cell proliferation in these cell lines, recombinant mouse EGF was added at 2 ng/mL. This is roughly 500-times the reported EC_50 _for growth stimulation and 20-times higher than levels found in the BALF from tumor-bearing animals (Figure 6A). EGF had no significant effect on tumor cell proliferation when added alone, and did not significantly affect the ability of either IGF-1 or MØCM to stimulate neoplastic growth (Figure 7E, F). This is not surprising in view of recent studies showing that EGFR inhibitors do not inhibit growth of lung cells with *KRAS *mutations [40].

As IGF-1 was sufficient to induce neoplastic proliferation, we determined whether the IGF-1 and MØCM growth effects were additive. A dose of 50 ng/ml IGF-1 stimulated neoplastic growth to a similar extent as MØCM (Figure 7A-D); 2 ng/mL IGF is the reported EC_50 _for IGF-1 stimulated proliferation *in vitro *as well as the concentration detected in the BALF of tumor-bearing mice *in vivo *(Figure 6A). IGF-1 dose-dependently stimulated the proliferation of both LM2 and JF32 cells, and augmented the growth-stimulating effects of MØCM when added in combination. To determine if IGF-1R signaling mediates both IGF-1 and MØCM stimulation, lung cancer cells were pre-treated with vehicle or 5 μM NVP-AEW541 (- or +, respectively), and cell numbers determined as indicated. IGF-1 and MØCM each significantly increased cell numbers after 48 and 72 hrs, while pharmacological inhibition of IGF-1R signaling blocked IGF-1 and MØCM growth effects in both neoplastic lines (Figure 7G, H). Parallel comparison of MTS values indicated a highly significant correlation between live cell numbers and relative MTS scores (r^2 ^= 0.7912 and 0.8201 for LM2 and JF32, respectively, p < 0.0001, data not shown). Furthermore, both IGF-1 and MØCM increased the fraction of BrdU^+ ^LM2 cells 12-24 hrs after treatment, corresponding with significantly increased cell numbers (data not shown). These observations suggest that IGF-1, but not EGF, plays a major role in macrophage stimulated neoplastic growth *in vitro*, consistent with the elevated IGF-1 levels observed in lung-tumor bearing animals *in vivo*.

### MØCM stimulation of neoplastic growth is diminished when IGF-1 content is decreased

**Figure 8:**
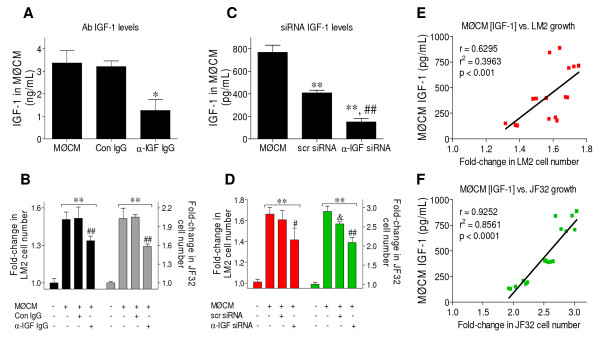
**MØCM IGF-1 content regulates the proliferation of treated neoplastic cells**. *A-B: *Macrophage-conditioned media (MØCM) was concentrated and immunoprecipitated against isotype control (Con IgG) or α-mIGF-1 IgG antibodies (α-IGF IgG). *(A) *Media IGF-1 content was analyzed by ELISA, and *(B) *LM2 or JF32 cells were cultured in serum-free media with 0.5% BSA, with or without the MØCM generated in (A). Relative growth was determined by MTS, and mean + SD plotted as fold-change from control, which was normalized to 1.0. Data was pooled from at least 3 independent experiments. * *P *< 0.01 vs. Con IgG; ** *P *< 0.001 versus untreated cells, and ## *P *< 0.001 versus the Con IgG group by 1-way ANOVA. *C-D: (C) *IGF-1 concentration in media conditioned by macrophages pre-treated with scrambled control siRNA (scr siRNA) or α-IGF-1 siRNA construct, vs. naïve MØCM, was determined by ELISA; *(D) *LM2 or JF32 cells were cultured and relative cell number determined as in (C). (C) ** *P *< 0.001 vs. naïve MØCM and ## *P *< 0.001 versus the scr-siRNA by 1-way ANOVA; (D) ** *P *< 0.001 vs. untreated cells; # *P *< 0.05 and ## *P *< 0.001 vs. the scr siRNA group; and &*P *< 0.001 vs. the MØCM group by 1-way ANOVA. *E-F: *Pearson correlation and linear regression of MØCM IGF-1 levels from (C) vs. the fold-change in LM2 or JF32 cell number from (D) after MØCM addition; (E) LM2 *P *< 0.001 and (F) JF32 *P *< 0.0001.

In order to determine if IGF-1 was a molecular mediator directly responsible for growth stimulated by MØCM, we decreased MØCM IGF-1 content through two independent avenues: immuno-depletion and siRNA interference. MØCM was concentrated to contain ~3.5 ng/mL IGF-1, and then incubated with control IgG (Con IgG) or α-IGF-1 IgG coated resin, as described [39]. This procedure successfully decreased MØCM IGF-1 levels to 40-50% of control (Figure 8A). When this IGF-1 depleted media was added to LM2 and JF32 cells, growth stimulation was significantly decreased compared to untreated MØCM or IgG controls, which were identical (Figure 8B). In addition, MH-S macrophage IGF-1 secretion was interrupted by transfection with scrambled control (scr siRNA) or siRNA constructs designed against mouse IGF-1 (α-IGF siRNA). One α-IGF siRNA construct was more effective than the scr siRNA, and significantly decreased MØCM IGF-1 levels to 25% of control (Figure 8C). The scr siRNA construct decreased macrophage IGF-1 secretion to a lesser extent (Figure 8C). Transfection reagents and conditions were chosen to minimize cellular toxicity, and media IGF-1 content significantly decreased when normalized to MH-S viability (media IGF-1/MTS relative viability, data not shown). Neoplastic growth reflected the level of IGF-1 in the media conditioned by siRNA-treated macrophages, with all three groups differing significantly in JF32 cells (Figure 8D). While scr siRNA-treated media did not significantly lower LM2 cell growth, the correlation of media IGF-1 levels vs. LM2 proliferation was highly significant, as in JF32 cells (Figure 8D-F). Taken together, these experiments demonstrate that IGF-1 is responsible for the majority of neoplastic growth stimulated by MØCM.

### Combined MEK and PI3K inhibition blocks IGF-1 and MØCM induced neoplastic proliferation by decreasing cyclin D1 expression

**Figure 9:**
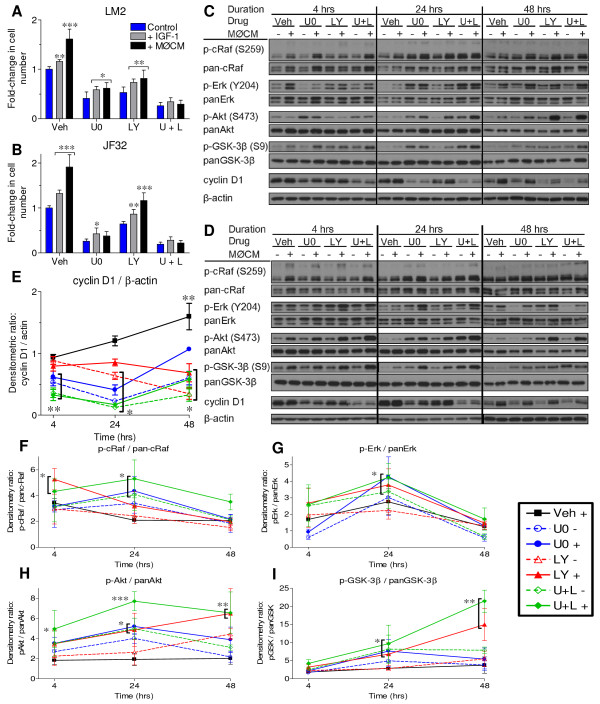
**MEK and PI3K inhibition blocks MØCM induced growth, but dissociates kinase activity from cyclin D1 expression**. *A-B: (A) *LM2 or *(B) *JF32 cells were cultured alone (Control), with 2 ng/mL mIGF-1 (+ IGF-1) or with media conditioned by MH-S macrophages (+ MØCM), and treated with 0.05% DMSO (vehicle, "Veh"), 5 μM U0126 (U0), 10 μM LY294002 (LY) or both agents together (U + L). Relative cell number was determined by MTS, and mean + SD plotted as fold-change from vehicle control, which was normalized to 1.0. Data was pooled from at least 3 independent experiments. * *P *< 0.05, ** *P *< 0.01 and *** *P *< 0.001 versus control for each treatment by 2-way ANOVA. *C-D: *LM2 *(C) *or JF32 *(D) *cells were cultured as described above, and cell homogenates probed for protein expression as described. Western blot images are representative of two independent experiments. *E-I: *densitometry data from all western blot replicates was collected from both LM2 and JF32 cell lines, combined, and presented as fold change (mean ± SEM) from untreated vehicle control lanes at each time point ("Veh -" group normalized to 1, not shown). The legend colors indicate drug treatment groups, followed by a "- or +" to indicate the absence or presence of MØCM, as in *(A-B)*. * *P *< 0.05, ** *P *< 0.01 and *** *P *< 0.001 versus control (Veh -) cells at each time point by 2-way ANOVA.

IGF-1 stimulated neoplastic proliferation and mediated a significant portion of macrophage-induced tumor cell growth in culture. To determine if MØCM and/or IGF-1 were similarly blocked by MEK and PI3K inhibition, LM2 and JF32 cells were treated with combinations of MEK and/or PI3K inhibitors, in the presence of IGF-1 or MØCM. Analogous to previous results with macrophage co-culture, growth stimulated by either IGF-1 or MØCM was blocked by combined inhibition of MEK and PI3K, to a greater extent than either pathway by itself (Figure 9A, B). Consistent with the proliferation results, cyclin D1 content (a biochemical correlate of lung cell proliferation [41]) was reduced by these inhibitors (Figure 9C-E).

MØCM induced early increases in cRaf, Akt and GSK-3β phosphorylation, and Erk1/2 phosphorylation peaked at 24 hrs (Figure 9C, D). In both LM2 and JF32 cells, increased Akt phosphorylation corresponded to more phosphorylation of the Akt substrate, pGSK-3β (Figure 9H and 9I). Phospho-cRaf levels, another marker of Akt activity, also increased in concert with heightened increased Akt activity from 4-24 hrs; although p-cRaf abruptly dropped at 48 hrs, pAkt and pGSK-3β levels remained highly elevated (Figure 9F, H and 9I).

We observed reciprocal changes in the Erk and Akt pathways in response to their respective enzyme inhibitors. In LM2 cells, MEK inhibition (by U0126) suppressed early Erk1/2 phosphorylation while p-Akt levels increased. Conversely, PI3K inhibition (by LY294002) increased basal p-Erk1/2 levels at the expense of p-Akt (4 hrs time point; Figure 9C). MEK inhibition raised p-Erk1/2 and total Erk1/2 levels at 24 and 48 hrs, while PI3K inhibition caused a compensatory increase in cellular p-Akt levels from 24-48 hrs. JF32 cell growth was also suppressed by each drug; although MEK inhibition did not affect p-Erk1/2 levels at 4 hrs, p-Erk1/2 levels decreased at 48 hrs (Figure 9D). PI3K inhibition stimulated Erk1/2 phosphorylation from 4-24 hrs, and increased Akt phosphorylation throughout the treatment time-course (Figure 9G, H).

While each inhibitor decreased basal proliferation rates (Figure 9A, B), combinations of kinase inhibitors and MØCM increased cRaf, Erk1/2, Akt and GSK-3β phosphorylation in an additive manner, with the highest levels observed in cells treated with both kinase inhibitors and MØCM ("U+L +", Figure 9C-I). Total and p-cRaf, p-Akt and p-GSK-3β were each significantly higher after 4-24 hrs of treatment in all groups receiving any combination of drug and MØCM, and p-Erk1/2 levels spiked after 24 hrs of treatment (Figure 9F-I). Either inhibitor alone partially prevented the increase in cyclin D1 in cells treated with MØCM; cells receiving both inhibitors had the lowest cyclin D1 levels and were unresponsive to MØCM-induced growth (Figure 9E). Taken together, MØCM-induced neoplastic Akt and Erk1/2 phosphorylation was magnified several-fold by inhibitor treatment, dissociating kinase activity from proliferation in drug-treated cells; however, cyclin D1 levels were suppressed by either drug alone, which corresponded to decreased cell proliferation.

**Figure 10:**
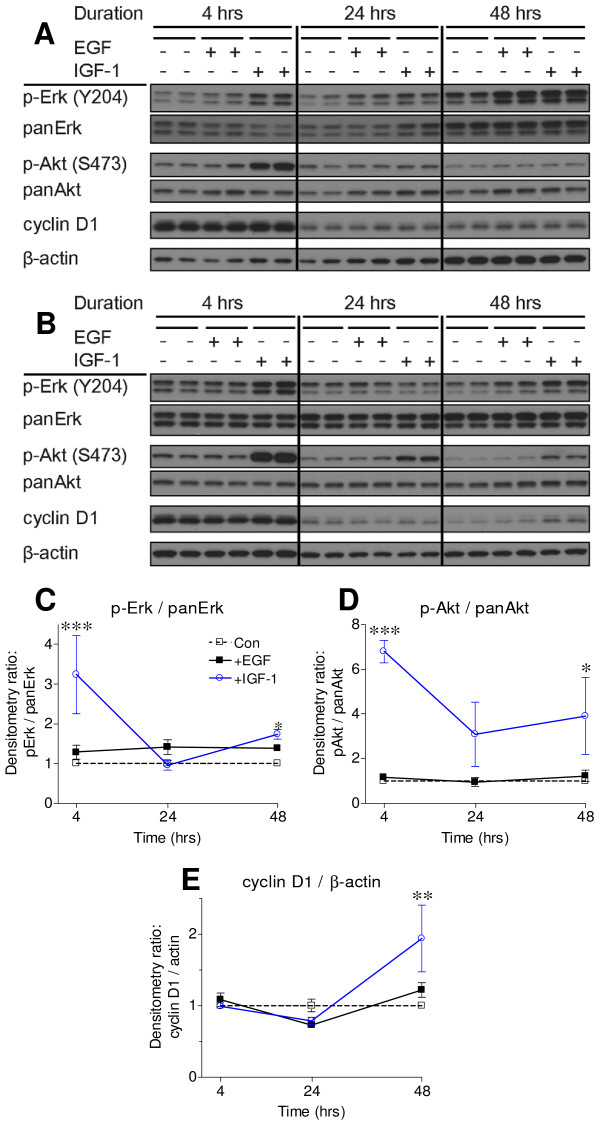
**IGF-1 stimulates Erk and Akt activities and elevates cyclin D1 expression**. *A-B: (A) *LM2 or *(B) *JF32 cells were cultured in serum-free media, with or without 2 ng/mL mIGF-1 or mEGF, and cell homogenates were probed for protein expression. *C-E: *densitometry from western blot replicates from LM2 and JF32 cell lines was combined, and presented as fold change (mean ± SEM) from untreated vehicle control lanes at each time point. * *P *< 0.05, ** *P *< 0.01 and *** *P *< 0.001 versus control (Veh -) cells at each time point by 2-way ANOVA.

As with MØCM, IGF-1 stimulated both Akt and Erk1/2 activities. Kinase activation was greatest within 4 hrs of treatment, and remained elevated 48 hrs later, corresponding with increased cyclin D1 expression (Figure 10A-E). When treated with 2 ng/mL EGF, a concentration 1,000-times higher than the amount of EGF in cell-conditioned media and 40-times higher than what is detected in BAL fluid, Erk1/2 activity was not significantly elevated and Akt levels were unaffected (Figure 10C, D). EGF may partially stimulate Erk1/2 activity at supra-physiological levels, but this was not sufficient to stimulate cellular growth. When administered at cell-and tissue-relevant levels, IGF-1 stimulated both Erk1/2 and Akt activation, elevated cellular cyclin D1 content, and induced neoplastic proliferation.

## Discussion

Our results suggest that inflammatory macrophages directly stimulate lung tumor growth through increased local production of IGF-1. We show that both naïve and tumor-educated primary lung macrophages stimulate the proliferation of lung epithelial cells *in vitro*; recombinant IGF-1 recapitulates this effect, and the degree of macrophage-induced growth stimulation correlates with media IGF-1 levels. IL-4 stimulates primary lung macrophages to produce significantly more IGF-1 *in vitro*. Tumor-educated macrophages produce more IGF-1 on a per-cell basis than naïve BAL macrophages, consistent with the elevated levels of T_H_2-like cytokines reported in the lung tumor microenvironment. Secretory products of macrophages stimulate neoplastic Erk1/2 and Akt activity, increase cyclin D1 expression, and accelerate growth. Both macrophage conditioned media (MØCM) and recombinant IGF-1 stimulate neoplastic proliferation, which can be ablated by the combined inhibition of MEK and PI3K.

Sustained changes in macrophage phenotype exacerbate several lung diseases, and alternative macrophage activation is an early event in lung tumorigenesis [6,42,43]. T_H_2 cytokine levels rise in AC-bearing mice and human NSCLC patients, and alternative activation resulting from T_H_2-like cytokines increases IGF-1 macrophage production [11,38,39,44]. Selectively removing alternatively-activated macrophages reduced lung tumor colonization in mice [45]. In agreement with these reports, we show that *in vitro *IL-4 stimulation enhanced IGF-1 production by primary BAL macrophages. Tumor-educated BAL macrophages produced significantly more IGF-1 than naïve macrophages, both basally and in response to IL-4 stimulation. We previously found that lung tumors recruit increasing numbers of macrophages to the alveolar space [6]. Therefore, the lung tumor environment contains not only more macrophages, but macrophages with heightened IGF-1 production. Consistent with this conclusion, BALF IGF-1 levels were > 3-fold higher in lung tumor-bearing mice compared to naïve littermates (Figure 6A-B).

While the role of primary lung macrophages in mediating lung cancer proliferation has not been previously examined, the effects of co-cultured stromal cell types on a *Kras *mutant mouse lung AC cell line (LKR-13) was recently reported [18]. When cultured with media conditioned by MH-S cells, proliferation of AC cells increased significantly, in agreement with our observations. This study focused on the migration resulting from the increased CXCL1 (KC) and IL-18 observed under co-culture conditions, and did not determine if exogenous KC or IL-18 stimulated neoplastic proliferation. They also found that MH-S conditioned media had no effect on neoplastic colony formation in soft agar, while we describe the potent stimulation of anchorage-independent growth of two *Kras *mutant lung tumor-derived cell lines, using two independent assays (Figure 1). By fractionating MØCM, we demonstrate that the factors responsible for stimulating neoplastic proliferation are 7-11 kDa, making IL-18 (18 kDa) an unlikely candidate. KC, on the other hand, is a potent 8 kDa chemokine. Based on molecular weight alone, we cannot rule out KC as contributing to the increased growth caused by MØCM; however, several lines of evidence make this unlikely. First, both MH-S and primary naïve BAL macrophages stimulate neoplastic proliferation, but KC was undetectable in media conditioned by MH-S macrophages [18] or primary BAL macrophages isolated from naïve or lung-tumor bearing animals (data not shown). Second, unlike IGF-1, KC expression does not increase in alternatively activated macrophages [11]; alternative activation increases IGF-1 production, and this stimulates neoplastic proliferation. Lastly, although Zhong, et. al. examined an exhaustive array of cytokines, they did not measure IGF-1 [18]; thus, they did not evaluate the role of IGF-1 in mediating the effects observed in their co-culture model. Our observations of lung macrophages complement previous reports regarding stromal cell stimulation of neoplastic growth and invasion, and expand upon them to demonstrate that macrophage-derived IGF-1 accelerates neoplastic lung cell growth *in vitro*. Macrophage IGF-1 may thus have a pathological role in lung cancer.

Direct connections between lung macrophages and AC progression *in vivo *are less clear than the well-described interactions between macrophages and breast cancer cells [14,16], or osteoclasts and oncolytic breast cancer metastases [46]. Lung tumor cells over-expressing IL-1β enhanced macrophage recruitment and tumor angiogenesis when implanted into syngeneic mice [19]. In our studies, BALF CSF-1 levels were nearly undetectable while IL-1β levels were significantly higher in tumor-bearing lungs vs. naïve (data not shown). However, recombinant IL-1β did not affect the proliferation of neoplastic lung epithelial cells *in vitro*, either alone or in combination with IGF-1. IL-1β also did not significantly affect IGF-1 production by MH-S macrophages (data not shown). Although not responsible for the macrophage-induced neoplastic proliferation observed in our studies, IL-1β stimulated macrophages produce more pro-angiogenic factors, and this interleukin may contribute to the increased numbers of macrophages in tumor-bearing lungs [6,10,19].

In lung cancer therapy, anti-angiogenic or anti-inflammatory agents show widespread efficacy across many cancer types, while inhibition of the EGF receptor (EGFR) is mainly effective in the NSCLC sub-population containing activating EGFR mutations [40,47]. EGFR-mutant lung cancers eventually become resistant to anti-EGFR therapies, and then progress rapidly [12,40]. One proposed mechanism for lung cancer resistance to anti-EGFR therapy is the increased expression of other EGFR family receptors and/or the IGF-1 receptor [48]. Similar to the well-described hetero-dimerization among the EGF receptor family, IGF-1R can form functional complexes with EGFR [12,48]. Unlike IGF-1R, EGFR can be stimulated by numerous EGF-like factors, which macrophages produce in a tissue and disease-specific manner [10,14,49]. However, we show that: 1) BALF EGF levels are very low and do not differ between naïve and tumor-bearing lungs; 2) macrophages produce trace amounts of EGF *in vitro*; and 3) EGF does not stimulate neoplastic lung proliferation either alone or in combination with IGF-1 or MØCM (Figures 6, 7 and data not shown). Combined, these observations indicate that EGF is not involved in the macrophage-stimulation of pulmonary epithelial growth *in vitro*, and argue against significant lung macrophage EGF production *in vivo*. The increased EGFR phosphorylation in primary mouse lung tumors bearing *Kras *mutations that we previously reported could result from IGF-1R/EGFR coupling and trans-activation after IGF-1 stimulation [48,50]. Mutations in *EGFR *and *KRAS *are mutually exclusive in both human and murine NSCLC, and EGF stimulation would not be expected drive *Kras *mutant models of lung cancer [23,40,50]. A requirement for the IGF-1 receptor in mediating lung cancer growth is consistent with other reports that IGF-1 stimulates rapid anchorage-independent growth *in vitro*, while IGF-1R inhibition slows tumor growth in both animal xenograft studies [27] and human clinical trials [12].

IGF-1R signals through numerous downstream pathways in which the intracellular kinases Erk1/2 and Akt are frequently activated [12,48]. We have previously determined that MEK inhibition induces a potent G1 phase arrest in neoplastic lung cell cycle progression *in vitro *[51], and others have determined that blocking both MEK and PI3K slows lung tumor growth *in vivo *[25]. We show herein that MØCM-stimulated neoplastic proliferation significantly increases cyclin D1 expression, which is abrogated by the combined inhibition of both MEK and PI3K. Sole inhibition of either MEK or PI3K partially limits macrophage stimulation of LM2 and JF32 growth to slightly different extents. While MØCM modestly increases Erk1/2 and Akt activity, long-term MEK and PI3K inhibition strikingly stimulates both kinases in an additive manner with conditioned media treatment. This increased kinase activity resulting from MEK and PI3K inhibition, however, is no longer associated with changes in cyclin D1, as combined inhibition resulted in the highest levels of Akt activity, but lowest levels of cyclin D1 expression ("U + L"; Figure 9E and 9H). Compensatory Akt or Erk activation in response to upstream kinase inhibition is consistent with the extensive cross-talk that exists among MAPK pathways, where inhibition of any single mediator results in exaggerated and/or sustained signaling through an alternate pathway [32,34,52,53]. Indeed, when the MEK pathway was inhibited in LM2 cells, early p-Akt activity increased, while PI3K inhibition increased p-Erk1/2 (4 hrs: Figure 9C). Akt is hyper-phosphorylated with 24 hrs of treatment with either MEK or PI3K inhibitor, and this hyper-activated Akt sustains 5-10 higher levels of p-GSK-3β and p-cRaf for at least 48 hrs. Erk1/2 phosphorylation is also stimulated by drug treatment, which peaks at 24 hrs and rapidly declines by 48 hrs. Consistent with our observations, continuous hyper-activation of Akt or Erk1/2 induces cytostasis or even apoptosis in some tissues, while more modest Erk1/2 activation drives *Kras *mutant tumor cell proliferation [34,40,51]. While our studies demonstrate that MØCM and IGF-1 stimulated neoplastic growth is affected similarly by MEK and PI3K inhibition, further studies in genetically-silenced or kinase-mutant cell lines are required to determine the discrete cellular mechanisms necessary for growth factor-stimulated neoplastic proliferation.

*Kras *mutant lung tumors may rely on growth factor stimulation *in vivo *to regulate binding partner localization and activation. Kras can only efficiently trigger proliferation by recruiting partner kinases like cytosolic Raf (cRaf/Raf-1) to the plasma membrane, where cRaf is phosphorylated and activated by ligand-bound growth factor receptors [33,54]. By phosphorylating mutant Kras-bound cRaf, growth factors can potently engage the ras-Raf signaling cascade, which deactivates slowly due to decreased GTPase activity of mutant Kras [24,54]. Akt phosphorylates cRaf at S259, which creates a binding domain for 14-3-3 protein family members [32,33]. 14-3-3 binding is required to inactivate cRaf, as p-S259 alone does not affect cRaf activity. However, mutant Kras can displace 14-3-3 from the p-S259 region of cRaf [33]. Thus, active Akt could phosphorylate and inactivate cRaf, leading to decreased Erk1/2 signaling, but cells with mutant Kras can bypass this regulatory mechanism and maintain high cRaf activity [33,54]. Consistent with these reports, we observe significant increases in neoplastic Akt, cRaf and Erk1/2 phosphorylation, suggesting that these *Kras *mutant cells bypass Akt-mediated MEK pathway inactivation (Figures 3 and 9). Due to the complex interactions between Erk and Akt, IGF-1 stimulated growth regulation in *Kras*-mutant NSCLC cells should be the subject of future investigation.

## Conclusions

In summary, we have identified IGF-1 as one factor produced by alveolar macrophages that directly stimulates neoplastic lung proliferation *in vitro*. These findings, in combination with correlations between macrophage numbers, activation state and IGF-1 levels *in vivo*, imply that IGF-1 mediates macrophage stimulation of NSCLC growth. This additional evidence links previous observations of macrophage depletion to tumor growth suppression. Macrophages are crucial for the progression of numerous cancers, including lung cancer, and IGF-1 has long been associated with resistance to chemotherapy and increased neoplastic proliferation.

Our results suggest that current anti-growth factor therapy could be augmented by removing the stromal source of neoplastic growth stimulation, in addition to blocking discrete elements of downstream signal transduction. This may be an effective strategy for the treatment of lung cancer and other diseases in which macrophage recruitment is associated with aberrant tissue proliferation.

## Methods

### Mice

Male A/J mice (4-6 wks old) were purchased from the Jackson Laboratory (Bar Harbor, ME), housed on hardwood bedding with 12 hr light/dark cycles, and fed Harlan Teklad 22/5 rodent chow *ad libitum *(Harlan, Madison, WI) at the Center for Comparative Medicine in the University of Colorado, Anschutz Medical Campus. Procedures were approved by the Institutional Animal Care and Use Committee of the University of Colorado.

### Isolation of lung protein exudates and alveolar macrophages

Primary alveolar macrophages and lung protein exudates were isolated by bronchoalveolar lavage (BAL) from male A/J mice 24-32 wks after a single i.p. injection of 10 mg/g ethyl carbamate (urethane; Alfa Aesar; Heysham, Lancashire. U.K.) or 0.9% NaCl vehicle control, as previously described [6,55]. This dose of urethane induces multiple lung tumors in A/J mice, which are mainly adenomas at 24 wks and progress to AC from 24-42 wks. BAL macrophages from control animals are considered "naïve", while macrophages isolated from lung tumor-bearing mice are "tumor-educated" [14].

### Generation of JF32 cells from primary lung tumor isolates

Thirty-two wks after urethane injection, male A/J lung tumors were resected from the lung under a dissecting microscope. Fifty mg of tumor tissue was placed onto a sterile Pyrex petri dish, finely chopped in 0.2 mL PBS with a sterile razor, and the resulting suspension added to a Krebs-Ringer buffered solution containing 10 U/mL Dispase 10 U/mL collagenase I (Worthington, Lakewood, NJ.). The tumor suspension was digested with agitation for 60 min. at 37°C, after which digestion was terminated by adding an equal volume of 20 mM EDTA. The tumor suspension was then passed twice through a 20 ga syringe needle, and filtered to create a single cell suspension of tumor cells, as described for the isolation of primary Clara cells [56,57]. These tumor cells were washed 3-times in 10% FBS (Hyclone; Logan, UT) MEM-α (Invitrogen; Carlsbad, CA), collected by centrifugation, and their viability determined by trypan-blue (Sigma-Aldrich; St. Louis, MO) exclusion using a hemocytometer. The primary tumor isolates were > 90% viable by this method. Twenty thousand cells per well were plated in 1% FBS MEM-α on Matrigel-coated 6-well plates (Fisher; Waltham, MA). The primary tumor cell cultures were maintained for 4 weeks, and MEM-α media containing 1% FBS changed once weekly. For three weeks, there was little morphological change in colony size or number, and then actively proliferating colonies were observed. Two adherent colonies were removed (using a trimmed pipette tip), designated "JF32a" and "JF32b", plated onto standard 100 mm tissue culture-treated plates, and cultured as described below. Exon 2 of the *Kras *gene was sequenced as previously described [50], and Q61R *Kras *mutations detected in both JF32a and b, consistent with our previously published report of *Kras *mutation incidence in urethane-induced mouse lung tumors [50].

### Cell culture

The non-tumorigenic, mouse type II pneumocyte-derived epithelial cell line (E10) was used to represent non-transformed lung epithelium *in vitro*. To study the interactions of tumor cells with macrophages, three neoplastic mouse lung cell lines were used: the newly generated JF32a cells (hereafter "JF32"); LM2, previously derived from a urethane induced lung tumor in A/J mice; and E9, a spontaneous transformant of E10 cells [57]. Culture of all cell lines was previously described; JF32 cells were maintained like the LM2 cell line [21,57]. To study the *in vitro *effects of immune mediators on epithelial cells, MH-S macrophages (American Type Culture Collection; Manassas, VA), an alveolar macrophage cell line isolated from a BALB/c mouse [58], or primary BAL macrophages were used. All macrophages were maintained in RPMI 1640 (Invitrogen) according to ATCC guidelines for the MH-S cell line. All cells were cultured in a humidified atmosphere of 5% CO_2 _in ambient air at 37°C, and routinely screened for *Mycoplasma *contamination (MD Biosciences Inc; St. Paul, MN). Where indicated, 2-50 ng/mL recombinant mouse IGF-1 (R&D Systems, Inc.; Minneapolis, MN.) and/or EGF (eBioscience, Inc.; San Diego, CA) were added to epithelial cultures.

### Anchorage-independent culture

LM2 and JF32 cells were suspended in 0.5% low-melting point agarose (Ultra-pure LMP agarose, Invitrogen) in MEM-α media containing 0.5% BSA (Sigma), and plated at 1,000 cells/well into 12 well plates with a pre-coated base layer of 1% agar (Sigma), and a top layer of 0.75% LMP agarose. Once weekly, cells were fed with 0.5 mL MEM-α + 0.5% BSA or macrophage-conditioned media (with 0.5% BSA, described below). After 5-6 wks of growth, colony number (clumps of ≥ 10 cells) was determined under 20× magnification with a bright-field inverted microscope (Nikon Instruments Inc, Melville, NY). Alternatively, neoplastic cells were suspended in MEM-α media containing 0.5% BSA, and plated at 3,000 cells/well onto ultra-low attachment 6-well culture plates (Costar, Corning, NY). Cells were fed once weekly with 1 mL MEM-α + 0.5% BSA or macrophage-conditioned media. After 3 wks, the contents of each well were removed with a pipette, and cells pelleted by 5 min. centrifugation at 600 × g. Cells were resuspended in 1.5 mL Accutase (Sigma), and incubated for 20 min. at 37°C to create a single-cell suspension. Equal volumes of cell suspension were added to 0.4% Trypan-blue solution (Sigma), and live vs. dead cells ascertained using a hemocytometer.

### Macrophage co-culture and conditioned media

Epithelial cell lines were plated onto tissue culture-treated plates (Costar). Macrophages were plated onto 0.4 μm pore Transwell inserts (Becton Dickinson, Franklin Lakes, NJ) to allow diffusible signals to exchange during co-culture while preventing physical contact. Epithelial cells and macrophages were plated separately in media containing 10% FBS and allowed to equilibrate overnight. All co-culture systems consisted of macrophages co-incubated with epithelial cells at a 1:5, macrophage to epithelial cell ratio (2-4 wells/condition). Co-culture was initiated by replacing the original media with fresh serum-free MEM-α + 1% BSA media (SF MEM-α), and inserting the macrophage-containing Transwells into wells containing epithelial cells. To study the direct effects of macrophage-derived molecules on epithelial cells, media conditioned by primary BAL macrophages was generated by culturing 100,000 macrophages in 24-well plates in 1 mL media for 24 hrs. This macrophage-conditioned media (MØCM) was then added to epithelial cell-containing wells at a 1:1 ratio with fresh media. For additional experimental analysis, SF MEM-α media was conditioned by MH-S macrophages at 1 million macrophages/mL for 24 hrs, and added to cells as above.

### Conditioned media fractionation and IGF-1 immuno-depletion

MØCM from MH-S macrophages was collected and filtered through Microcon 0.5 mL volume spin filters (Millipore, Bellerica, MA), with molecular-weight cut-offs (m.w.c.o.) of 3, 10 and 30 kDa, as indicated. Each column was rinsed 2× with PBS, and then 500 μL of MØCM or control SF MEM-α media applied and columns centrifuged at 11,000 × g @ 10°C until only ~50 μL remained. The concentrated media was removed and added to LM2 containing wells in 500 μL of fresh SF MEM-α. IGF-1 was depleted from MØCM following the method described by Wynes, et. al., with several modifications [39]. Conditioned media was first concentrated 4-times against a 3,000 kDa m.w.c.o. Amicon filter using a nitrogen pressure filtration chamber (Millipore) to yield a final IGF-1 concentration of 3-4 ng/mL. This MØCM concentrate was rotated for 2 hrs with 6 μg of α-mIGF-1 IgG antibodies, consisting of a 1:1:1 w/w ratio of: MAB791, AF791 (R&D systems, Inc) and sc-1422 (Santa Cruz Biotechnology, Inc, Santa Cruz, CA). As an IgG control, 6 μg of goat IgG α-COX-1 antibody (sc-1754, Santa Cruz) was used. Fifty μL of protein G-coated magnetic resin, prepared and washed as directed (DynaMax; Invitrogen), was added to the media + antibody solution, and rotated for 1 hr. The resin was separated from the solution with a Dynal bench-top magnet (Invitrogen) and discarded, while the MØCM was transferred to a sterile eppendorf tube. This process was repeated with fresh antibody prior to cell treatment.

### MH-S siRNA transfection

MH-S macrophages were transfected with siRNA targeted against murine IGF-1 according to manufacturer instructions for murine J774.1 macrophage transfection (Qiagen Inc, Valencia, CA), and then optimized for MH-S transfection as described below. Three α-IGF-1 siRNA constructs, SI01073996, SI01073982 and SI01073989 (Qiagen; http://www.qiagen.com/geneglobe), were evaluated for IGF-1 knockdown, as determined by IGF-1 levels in conditioned media. Knockdown efficiency was compared against naïve (untransfected) and AllStars negative control (scrambled siRNA) transfected cells; the AllStars negative control has no known homology to any mammalian gene (Qiagen). Constructs "...96" and "...82" were no more effective than the negative control, while "...89" (hereafter referred to as "α-IGF-1 siRNA") effectively knocked down IGF-1 release into culture media. The transfection reagent HiPerFect (Qiagen) exhibited low toxicity and was used to establish transfection conditions that maintained ≥ 80% viability in transfected cells vs. naïve. In brief, 150,000 MH-S macrophages/well were suspended in 200 μL of 10% FCS supplemented RPMI in 24-well plates and allowed to incubate as described above for 1-2 hrs. For each well, siRNA (1.5 μL of a 20 μM stock solution) was added to 100 μL of serum-free RPMI and vortexed prior to addition of 4.5 μL HiPerFect transfection reagent. After 4 hrs, 150 μL of 10% FCS RPMI was added; 12 hrs later another 150 μL of 10% FCS RPMI was added. After 48 hrs, the transfection media was removed and replaced with SF MEM-α + 0.5% BSA, which MH-S macrophages conditioned for 24 hrs. Successful IGF-1 depletion was monitored by ELISA, as described.

### Cell proliferation and viability

Relative cell number was determined by 3-(4,5-dimethylthiazol-2-yl)-5-(3-carboxymethoxyphenyl)-2-(4-sulfophenyl)-2H-tetrazolium assay (MTS;CellTiter 96^® ^One AQ_ueous_, Promega; Madison, WI) according to manufacturer's instructions, and measured spectrophotometrically at Abs_490 nm _(Vmax, Molecular Devices; Sunnyvale, CA). Additionally, cells were trypsinized, collected and counted with a hemocytometer after trypan-blue staining. All cell counts were normalized to control values for each cell line or treatment group, unless otherwise indicated.

### Determination of IGF-1 and EGF levels

IGF-1 and EGF were separately measured in biological samples by enzyme-linked immunosorbant assay (ELISA) in a 96-well format, according to the manufacturer's directions (R&D Systems, Inc.), and measured spectrophotometrically at Abs_450 nm _with wavelength correction set to Abs_550 nm_. All samples were diluted to be within the middle 60% of the 8-point standard curve, and concentrations calculated from log-transformed absorbance values, as recommended. In addition to standard curves, every plate contained an independent calibrator sample that tested within the range provided.

### Immunoblotting

Epithelial cell protein lysates were harvested after 48 hrs co-culture with MH-S macrophages (+), empty inserts (-, controls) or at the indicated time after conditioned media or growth factor addition (+), and compared to control wells without MØCM (-) as described [21,50], with the following modifications. Protein (5-10 ug/lane) was applied to 4-20% Tris-SDS Criterion gels (Bio-Rad, Hercules, CA), and separated proteins electro-transferred onto Immobilon-P PVDF membranes (Millipore). The membranes were blocked for 30 min. at room temperature in 100 mM Tris-buffered saline pH 7.4 with 0.1% Tween-20 (TBST) supplemented with the indicated concentration of non-fat dry milk, and incubated overnight at 4°C with primary antibodies diluted in blocking buffer with milk or bovine serum albumin (BSA; Sigma), as described in Table 1[51]. After washing, blots were incubated with horseradish-peroxidase conjugated secondary antibodies at the indicated dilution for 1 hr at room temperature (Table 1), and protein bands were visualized by chemiluminescence on X-ray film as previously described [50]. Antibodies against phospho-specific proteins were applied to freshly transferred membranes. After detection, membranes were stripped with 1 M Tris-HCl (pH 6.7) buffer containing 2% SDS and 0.86% 2-mercaptoethanol (Fisher) in a 50°C hybridization oven for 60 min., and probed with antibodies against total protein levels as indicated. Equal protein loading was confirmed by β-actin levels and Coomassie gel staining (Fisher). Band density was quantified by Un-Scan-It software (Silk Scientific, Orem, UT), and values normalized either to β-actin or relevant total protein bands on each PVDF membrane.

**Table 1:** Western blot antibody dilution

Antibody Target	Blocking/Antibody Dilution Buffer*	Primary Antibody Dilution†	Secondary Antibody Dilution	Antibody Source (1°/2°)‡
p-cRaf (S259)	TBST + 5% milk	1:2,000 (5% BSA)	1:10,000 goat anti-rabbit	CST/CST
c-Raf	TBST + 5% milk	1:1,000 (5% BSA)	1:10,000 goat anti-rabbit	CST/CST
pErk1/2 (Y204)	TBST + 2% milk	1:1,000	1:20,000 goat anti-mouse	S.C./S.C.
Erk1/2	TBST + 2% milk	1:50,000	1:40,000 goat anti-rabbit	S.C./S.C.
pAkt (S473)	TBST + 5% milk	1:4,000 (5% BSA)	1:10,000 goat anti-rabbit	CST/CST
Akt	TBST + 5% milk	1:10,000 (5% BSA)	1:20,000 goat anti-rabbit	CST/CST
pGSK-3β (S9)	TBST + 5% milk	1:1,000 (5% BSA)	1:10,000 goat anti-rabbit	CST/CST
panGSK-3β	TBST + 5% milk	1:2,000 (5% BSA)	1:10,000 goat anti-rabbit	CST/CST
Cyclin D1	TBST + 5% milk	1:2,000 (5% BSA)	1:10,000 goat anti-rabbit	CST/CST
β-actin	TBST + 2% milk	1:100,000	1:100,000 goat anti-mouse	Sigma/S.C.

*TBST, 100 mM Tris-buffered saline pH 7.4 with 0.1% Tween-20. † BSA, bovine serum albumin. ‡ Primary (1°) and secondary (2°) antibodies were obtained from Sigma, Santa Cruz Biotechnology, Inc. (S.C.; Santa Cruz, CA) and Cell Signaling Technology (CST).

### Drug treatment of cells

To selectively block activation of the Erk and Akt signaling pathways, selective inhibitors of MEK (U0126; Promega) and PI3K (LY294002; Cell Signaling Technology, Beverly, MA) were used at 5 μM and 10 μM, respectively [37]. Drugs were dissolved in DMSO in amber tubes immediately prior to use, and added in SF MEM-α to cells cultured alone, with MH-S macrophages, with MØCM, or with recombinant growth factors for 72 hrs. The concentration of DMSO in all experiments never exceeded the vehicle control of 0.05%. To selectively block IGF-1R signaling, NVP-AEW541 (Cayman Chemical Company; Ann Arbor, MI) was directly dissolved in to 0.5% BSA-supplemented MEM-α media, and added to cell-containing wells at a final concentration of 5 μM.

### Statistical analysis and estimation

To estimate the size of the MØCM factor responsible for stimulating neoplastic proliferation, we derived a function describing the extent of tumor cell growth (y) in terms of the size of molecules predicted to be contained in isolated fractions of conditioned media (x). The percent retention on size-exclusion columns (as fractions of the amounts loaded) vs. protein size (kDa) on each size m.w.c.o. column was provided by the manufacturer (Millpore) for six recombinant proteins of varying size. The resulting data set was plotted as percent retained vs. protein size, and the least complex "best-fit" equation was obtained using non-linear regression with SigmaPlot 2001 ver 7.101 (SysStat Software Inc., San Jose, CA). The extent that total, unfractionated MØCM stimulated LM2 proliferation was normalized to 100%. The extent that each retentate fraction stimulated LM2 growth was similarly calculated to determine the remaining percent of "growth stimulating ability" after filtration, as compared to unfractionated MØCM. The percent of growth stimulus remaining was equated to the percentage of protein standard retained, and the resulting protein size estimate calculated from the best-fit equation.

Densitometry measurements are presented as means ± SEM, and all other measurements as means ± SD [59]. Differences between conditions at specific time points were examined using Student's unpaired t-test when comparing only two groups, with Welch's correction for unequal variance when appropriate. For multiple comparisons, one-way and two-way ANOVA were used to compare interactions between co-culture conditions and proliferation rates as recommended [60,61]. The Bonferroni correction was used for multiple comparisons during ANOVA analysis. Correlation was performed using the Pearson method, and the corresponding linear regression plotted. All statistical tests for significance and correlation were performed using GraphPad Prism version 4.02 (GraphPad Software; San Diego, CA.); differences were considered statistically significant when *P *< 0.05.

## List of abbreviations

Akt: protein kinase B; AC: adenocarcinoma; BAL(F) bronhoalveolar lavage (fluid); cRaf: cytosolic Ras-1 (MAP3K); CSF-1: colony-stimulating factor 1; EGF: epidermal growth factor; ELISA: enzyme-linked immunosorbant assay; Erk1/2: extracellular-regulated kinase 1/2; GM-CSF: granulocyte-monocyte colony stimulating factor; GSK-3β: glycogen synthase kinase 3β; IGF-1: insulin-like factor 1; IL-1β: interleukin-1β; KC: keratinocyte-derived chemokine; MEK: mitogen-activated: extracellular regulated kinase (MAP2K); MØCM: macrophage-conditioned media; pan-: total levels; NSCLC: non-small cell lung cancer; PI3K: phosphoinositide 3-kinase.

## Competing interests

The authors declare that they have no competing interests.

## Authors' contributions

JF participated in the study conception and design, performed the experimental work, analyzed and interpreted the data, and wrote the manuscript. LN participated in the design of the study, experimental work and data interpretation. AM participated in the study conception, and contributed to data interpretation. All authors read, revised and approved the final manuscript.
